# Causal circuit tracing reveals distinct computational architectures in single-cell foundation models: inhibitory dominance, biological coherence, and cross-model convergence

**DOI:** 10.1093/bioinformatics/btag379

**Published:** 2026-06-15

**Authors:** Ihor Kendiukhov

**Affiliations:** Department of Computer Science, University of Tübingen, Tübingen, 72076, Germany

## Abstract

**Motivation:**

Sparse autoencoders (SAEs) decompose foundation-model activations into interpretable features, but the *model-internal causal* interactions between those features (i.e. what ablating one feature does to the others, as distinct from the biological causal structure of the underlying cells)—and how those model-internal relationships relate to biological structure—are uncharacterized in single-cell foundation models.

**Results:**

We introduce *model-internal causal circuit tracing*—zeroing one SAE feature at a source layer and measuring the resulting change in all downstream SAE features, for each of 120 source features—and apply it to Geneformer V2-316M and scGPT whole-human across four conditions (96 892 ablation-derived edges, 80 191 forward passes). On annotation-selected source features, edges share GO/KEGG/Reactome/STRING/TRRUST ontology terms at 50.9%–68.5%, a 2.9–6.2× enrichment over a configuration-preserving permutation null (P<.002); on 20 randomly sampled source features this attenuates to 21.5%–26.3%—still 2.5–3.1× above null—quantifying the annotation-selection contribution. *Inhibitory dominance* (fraction of ablation edges with d<0, i.e. source activation supports downstream target) is 65.5%–89.4%. scGPT produces larger raw per-edge effects (mean |d|=1.40 versus 1.05); after feature-share normalization, Geneformer is stronger (paired gene-pair ratio 0.64 on 33 301 shared pairs). Cross-model consensus yields 1142 architecture-invariant *domain pairs* (ordered pairs of GO biological-process categories “A→B” each connected by at least one ablation edge in both models; 10.6× enrichment over permutation null; *P*<.001). Circuit edge magnitude explains <1% of the variance in marginal driver-gene coexpression on the same cells ($R^{2}=0.010$, *n*=31 176): the graph encodes structure beyond bivariate correlation. Against a matched-cell-type ENCODE ChIP-seq prior, circuit-predicted transcription factor (TF)→target pairs are enriched 2.06× (Fisher OR 5.84), markedly higher than 1.12× against TRRUST; direct ChIP-seq-supported target pairs show 10–30× larger CRISPRi sign-bias-corrected excess than indirect pairs. Gene-level CRISPRi validation on Replogle K562 and the noncancer RPE1 arm (and a true primary-T-cell control from Shifrut E, Carnevale J, Tobin V *et al.* Genome-wide CRISPR screens in primary human T cells reveal key regulators of immune function. *Cell* 2018; 175: 1958–71.e15) after sign-bias correction shows excess over baseline of +0.03 and +0.35 percentage points on K562 and RPE1, respectively (baseline already 52%–56% from sign marginals); effect-magnitude Spearman correlations ρ≈0. Bootstrap and per-cell-type stability (*N*∈{50,100,200}; B cell, CD4${\;}^{+}$ T, macrophage) give Pearson r≥0.97 on shared edges with 100% sign agreement; edge Jaccard grows monotonically with sample size. The circuit graph is therefore highly reproducible as an effect-size map, cell type specific in edge identity, consistent with coexpression encoding, and weakly but detectably enriched for ChIP-seq-supported direct regulatory edges.

**Availability and implementation:**

https://github.com/Biodyn-AI/bio-sae-circuits (Python). Archival DOI: 10.5281/zenodo.19,633,166 (Zenodo).

## 1 Introduction

Transformer-based single-cell foundation models (scFMs), beginning with scBERT (Yang *et al.* 2022) and now including Geneformer (Theodoris *et al.* 2023), scGPT (Cui *et al.* 2024), scFoundation (Hao *et al.* 2024), and UCE (Rosen *et al.* 2023), encode cellular states in high-dimensional contextual representations. They are architectural relatives of the original Transformer (Vaswani *et al.* 2017) and of masked language models such as BERT (Devlin *et al.* 2019). They have been shown to produce gene embeddings that recover pathway structure, to predict cell-type labels, and to rank perturbation effects (Theodoris *et al.* 2023, Cui *et al.* 2024). Yet *what* biology these representations capture and *how* they internally transform gene-level input into contextual predictions remain largely opaque: any explanation beyond input–output behavior requires tools that expose the model’s internal computation.

Sparse autoencoders (SAEs) (Sharkey *et al.* 2022, Bricken *et al.* 2023, Huben *et al.* 2024) (online research reports: Sharkey *et al.* 2022, https://www.alignmentforum.org/posts/z6QQJbtpkEAX3Aojj/interim-research-report-taking-features-out-of-superposition; Bricken *et al.* 2023, https://transformer-circuits.pub/2023/monosemantic-features/) address the superposition hypothesis (Elhage *et al.* 2022, https://transformer-circuits.pub/2022/toy_model/index.html) by decomposing model activations into overcomplete dictionaries of monosemantic features, in the tradition of classical sparse coding (Olshausen and Field 1997). Recent scaling work (Templeton *et al.* 2024, https://transformer-circuits.pub/2024/scaling-monosemanticity/; Gao *et al.* 2025) has shown that SAEs recover interpretable features at language-model scale. Parallel mechanistic-interpretability work in language models has established a mathematical framework for transformer circuits (Elhage *et al.* 2021, https://transformer-circuits.pub/2021/framework/index.html), formalized the notion of a “circuit” via case-studies (Nanda *et al.* 2023, Wang *et al.* 2023) and via automated discovery methods (Conmy *et al.* 2023). SAE atlases for both Geneformer and scGPT have established that these models encode pathway membership, protein-interaction structure, and functional modules (Kendiukhov 2026). Those atlases, however, revealed *what* features exist and *where* in the network they are active; they did not reveal *how* features causally interact across network depth, which is the object of the present paper.

Causal-intervention methods—activation patching (Vig *et al.* 2020, Meng *et al.* 2022), causal abstraction (Geiger *et al.* 2021), and sparse-feature circuit discovery (Marks *et al.* 2025)—have proven powerful for dissecting information flow in language models, but have not been applied at the SAE-feature level in biological foundation models. Coactivation statistics (pointwise mutual information, a classical measure from corpus statistics; Manning and Schütze 1999) reveal which features tend to fire together but cannot distinguish correlation from causation or assign direction and magnitude to information flow.

Here, we introduce model-internal causal feature-to-feature circuit tracing: we ablate a source SAE feature at its layer and measure the resulting change in all downstream SAE feature activations across subsequent layers, yielding a directed graph of feature-to-feature dependencies. We apply this method to Geneformer V2-316M and scGPT whole-human under four conditions that independently vary cell-type input and SAE training data, and evaluate the resulting circuits against a falsifiable stack of biological priors.

### 1.1 Two notions of causality

We use “causal” in two senses. *Model-internal causality* refers to edges established by intervention on the model itself; *biological causality* refers to real regulatory edges in cells. These are related but not identical; we benchmark the former against the latter via ontology overlap, matched-cell-type ChIP-seq, and genome-scale CRISPRi, and report fold-enrichment over principled nulls.

### 1.2 Contribution

The methodological primitives—SAE feature decomposition, ablation, Cohen’s *d*—are not individually novel. Our distinct contributions are: (i) *scale*, 96 892 ablation-derived edges across 80 191 forward passes, enabling global statistical claims; (ii) *cross-model comparison* of two architecturally distinct biological foundation models under a common protocol; (iii) *input-condition factorization* (cell type × SAE training data) isolating SAE-dependent from data-dependent effects; (iv) a *composable, falsifiable validation stack* (ontology permutation nulls, matched cell-type ChIP-seq, direct versus indirect target CRISPRi partition, sign-bias-corrected perturbation accuracy, partial-correlation tests); and (v) *honest bias quantification*: for every headline coherence number, we report both the annotation-selected and the selection-free estimate.

## 2 Materials and methods

### 2.1 Models and SAEs

Geneformer V2-316M (18 transformer layers, $d_{\text{model}}=1152$, 18 attention heads, context window 4096 tokens; HuggingFace ctheodoris/Geneformer) and scGPT whole-human (12 transformer layers, d=512, 8 attention heads, 1200 padded positions). For scGPT we convert FlashMHA layers to standard MultiheadAttention (Wqkv.  →in_proj_ weight conversion) to enable forward hooks. SAE architecture: TopK (k=32) (Makhzani and Frey 2014, Gao *et al.* 2025) with 4× overcomplete dictionary (4608 features for Geneformer, 2048 for scGPT). *K562-only* Geneformer SAEs are trained on 1M subsampled K562 residual-stream positions per layer; *multi-tissue* Geneformer SAEs on 500K K562 + 500K Tabula Sapiens positions, at layers {0,5,11,17}. scGPT SAEs are trained on 3 561 832 Tabula Sapiens positions per layer. Ablation is implemented as a zero-clamp on the selected feature’s sparse coefficient followed by decoding and hidden-state replacement via torch.nn.Module forward hooks at the target layer.

### 2.2 Input data

K562 condition: 200 nontargeting control cells from the Replogle *et al.*’s (2022) genome-scale CRISPRi dataset. Tabula Sapiens: 200 cells stratified across immune (67 cells, 41 types), kidney (67 cells, 13 types), and lung (66 cells, 34 types), totalling 88 cell types. Cells are prefiltered for ≥500 detected genes and <10% mitochondrial content. Validation data: Replogle RPE1 noncancer arm (nontargeting controls n=11 485; 1446 targeted genes passing pool-level filter), and Shifrut *et al.*’s (2018) primary human CD8${\;}^{+}$ T-cell CRISPR-KO (n=52 236 cells, 30 683 nontargeting controls, 20 immune-checkpoint targets across two patients).

### 2.3 Causal tracing pipeline

**Figure 1:**
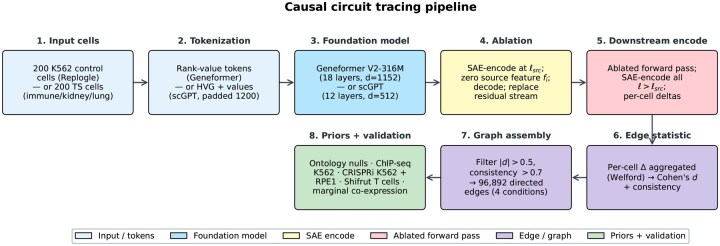
Model-internal causal circuit-tracing pipeline. Stages 1–5 (top row): cells are tokenized and passed through the foundation model, a source SAE feature $f_{i}$ at layer $\ell_{\text{src}}$ is zeroed in the sparse code, decoded, and substituted into the residual stream via a forward hook; downstream SAE features are reencoded on both clean and ablated passes. Stages 6–8 (bottom row, right to left): per-cell deltas are aggregated (Welford) into Cohen’s *d* and consistency; edges at |d|>0.5, consistency >0.7 form the 96 892-edge circuit graph across the four conditions; the graph is then evaluated against external biological priors.

For each of 120 source features (30 annotation-quality-selected features per layer at {0,5,11,15} for Geneformer, 90 at {0,4,8} for scGPT): (i) run a clean forward pass on each input cell; (ii) SAE-encode the source-layer residual stream; (iii) zero the source feature in the sparse code and decode to obtain a modified residual stream; (iv) run an ablated forward pass via a forward hook that replaces the original hidden state at the source layer with the modified one; (v) SAE-encode every downstream layer in both the clean and ablated passes; (vi) accumulate per-cell *deltas* of downstream sparse activations using Welford’s (1962) online algorithm across the 200 cells; (vii) finalize Cohen’s (1988)  $d=\bar{\Delta }/\sigma_{\Delta }$ and consistency (fraction of cells whose delta sign matches the mean-delta sign) per (source-feature, downstream-feature) pair (Fig. 1). An edge enters the circuit graph if |d|>0.5 and consistency >0.7. For efficiency, per-cell processing touches only feature indices active in that cell’s SAE encoding; inactive features contribute no information under zero-ablation, which eliminates a factor of ∼150 of unnecessary ablated forward passes on average.

### 2.4 Source-feature selection and bias

Annotation-selected features are scored by $\text{score}=10\cdot n_{\text{ontologies}}+n_{\text{annotations}}-{\;\text{log}\;}_{10}p_{\min}$ across GO BP, KEGG, Reactome, STRING, and TRRUST enrichments. To quantify selection bias, we additionally reran tracing on 20 randomly sampled source features from the annotation-qualified pool (two seeds).

### 2.5 Driver genes

For every SAE feature *f*, we define its *top-k driver genes* as the k=15 most activating genes: the genes whose rank-value (Geneformer) or HVG (scGPT) tokens yield the highest mean sparse-code magnitude for *f* across the training corpus, weighted by the corresponding SAE decoder-weight norm for the feature’s reconstruction contribution. Edges exported in the circuit graph carry source_genes and target_genes fields populated from these per-feature driver sets. All gene-pair analyses (E3/E4/E7 and the direct versus indirect partition) are built from the Cartesian product of source-driver × target-driver genes per edge, deduplicated across edges by |d|-weighted mean.

### 2.6 SAE reconstruction-error caveat

The ablation step decodes both the clean and zero-feature sparse codes through the same SAE decoder; the intervention applied to the residual stream is therefore $\Delta =\text{dec}(z_{\text{ablated}})-\text{dec}(z_{\text{clean}})$. Both decodes inherit the same reconstruction error, so this error cancels to first order in Δ. Residual second-order reconstruction noise contributes negligibly to Cohen’s *d* at N=200 (mean per-feature reconstruction $R^{2}>0.95$ in both SAE atlases; Kendiukhov 2026); we do not correct for it explicitly but flag it as an unavoidable methodological caveat for very small effect sizes.

### 2.7 Threshold justification

The |d|>0.5 cutoff is more conservative than a Benjamini–Hochberg-controlled false discovery rate (FDR) of 0.05 over ∼550 000 hypothesis tests: under a Welch-*t* null at n=200, critical |d| is 0.39. A magnitude sweep |d|∈{0.1,0.2,0.3,0.5,0.7,1.0} shows inhibitory% and shared-ontology% move ≤6 pp across {0.5,0.7,1.0}. The companion *consistency* threshold (>0.7) is the fraction of cells whose per-cell delta has the same sign as the mean delta; this removes edges whose sign is carried by only a small fraction of cells. Headline counts change by ≤8% when the consistency threshold is varied in {0.6,0.7,0.8} (Supplementary Material, available as supplementary data at *Bioinformatics* online).

### 2.8 Biological priors

(i) *Ontology coherence*: an edge has “shared ontology” if the intersection of its source and target feature annotation sets—pooled across Gene Ontology Biological Process (Ashburner *et al.* 2000), KEGG (Kanehisa and Goto 2000), Reactome (Jassal *et al.* 2020), STRING (protein–protein interaction confidence ≥0.7) (Szklarczyk *et al.* 2023), and TRRUST (Han *et al.* 2018)—is nonempty. Gene-set enrichment testing follows standard hypergeometric-with-multiple-testing protocols (Subramanian *et al.* 2005, Liberzon *et al.* 2015), with Benjamini–Hochberg false-discovery-rate control (Benjamini and Hochberg 1995). Null: configuration-preserving permutation that shuffles term sets across features of the same SAE layer (500 permutations). (ii) *Matched-cell-type ChIP-seq*: ENCODE (ENCODE Project Consortium 2012) 5-cell-line TF–target edges (184 TFs, 22 076 targets, 1 521 659 edges; K562 the largest contributor with 150/690 experiments); a K562-restricted subset filtered by wgEncodeRegTfbsClusteredInputsV3 retains 100 TFs and 943 160 edges. An alternative regulon prior is the DoRothEA resource (Garcia-Alonso *et al.* 2019), which aggregates TF–target evidence across multiple types. (iii) *CRISPRi perturbation*: the family of pooled CRISPR-single-cell screens pioneered by Dixit *et al.* (2016) and Adamson *et al.* (2016) and extended via CROP-seq (Datlinger *et al.* 2017); we use Replogle *et al.*’s (2022) K562 (nontargeting controls n=10 691) and the RPE1 noncancer arm of the same screen (n=11 485), plus Shifrut *et al.*’s (2018) primary human CD8${\;}^{+}$ T-cell CRISPR-KO. Per-target pseudobulk ${\;\text{log}\;}_{2}\text{FC}$ versus nontargeting controls. Guide efficacy filter: ${\;\text{log}\;}_{2}\text{FC}<-0.5$ and Welch *P*<.05 for CRISPRi; a KO-scale relaxation (${\;\text{log}\;}_{2}\text{FC}<-0.04$) is necessary for Shifrut because CRISPR-KO preserves transcript (median self ${\;\text{log}\;}_{2}\text{FC}=-0.03$ versus −1.5 for CRISPRi). (iv) *Marginal coexpression*: Pearson correlation between driver-gene pairs in 500 K562 nontargeting cells.

### 2.9 Sign-bias correction

Directional-accuracy numbers are biased by the near-identical sign marginals of predictions (∼85% negative, from inhibitory dominance) and observations (53%–59% negative). We report accuracy alongside a sign-bias null: P(pred<0)·P(obs<0)+P(pred>0)·P(obs>0). The informative quantity is *excess* over this null, not raw accuracy.

### 2.10 Stability

*Sample size*: L0 with 20 annotation-selected features at N∈{50,100,200}, same cell ordering. *Per-cell type*: 50 cells from a single immune type (B cell, CD4${\;}^{+}$  αβ T-cell, macrophage) with multitissue SAEs. Compared via edge Jaccard, Pearson r(d) on shared edges, and sign agreement.

### 2.11 Cell tokenization and preprocessing

For Geneformer, cells are tokenized via rank-value encoding (Theodoris *et al.* 2023): each gene’s expression is normalized by its corpus-wide median, and nonzero genes are ordered by the resulting relative-overexpression score to produce a rank-position sequence (max 2048 positions used here; Geneformer V2-316M’s architectural context window is 4096). For scGPT, cells are tokenized with continuous log normalized expression values, genes sorted by expression in descending order, and the sequence padded to 1200 positions. Upstream preprocessing uses scanpy (Wolf *et al.* 2018). Tokenizations are identical to the companion SAE-atlas pipeline. All model, SAE, and hook code is implemented in PyTorch (Paszke *et al.* 2019).

### 2.12 Permutation null construction

For the shared-ontology metric, we use a *configuration-preserving* null that shuffles annotation term sets among features within the same SAE layer, preserving per-layer term set size distributions. This breaks the specific feature→term pairing while maintaining the pool of ontology terms represented in each layer, controlling for the possibility that some layers are better annotated than others. We report empirical *P-*value from 500 permutations; for cross-model consensus, 1000 permutations on feature-correspondence shuffles. For the ChIP-seq enrichment, we use Fisher’s exact test against the TF×target background universe (184 TFs × 22 076 targets = 4 061 984 possible pairs; with 1 521 659 observed ChIP-seq edges the background density is 37.5%).

### 2.13 Availability

Code: https://github.com/Biodyn-AI/bio-sae-circuits; SAE atlases: https://github.com/Biodyn-AI/bio-sae; archival DOI for the frozen code release associated with this paper: 10.5281/zenodo.19,633,166 (Zenodo). All experiments run on a single Apple Silicon laptop (MPS backend); full K562/K562 tracing completes in 7.5 h, scGPT TS/Multi in 37 min; per-cell-type runs with multitissue SAEs complete in 10–13 min each. *Datasets and accessions*: Replogle *et al.*’s (2022) genome-scale CRISPRi, GEO GSE264667 (K562 essential Perturb-seq) and processed replogle_concat.h5ad; Shifrut *et al.*’s (2018) primary CD8${\;}^{+}$ T-cell CRISPR-KO, GEO GSE119450; Tabula Sapiens v1.0 (Tabula Sapiens Consortium 2022), figshare/CELL×GENE; ENCODE Uniform TFBS clusters V3 (accession wgEncodeRegTfbsClusteredV3) with InputsV3.tab cell-line metadata. Random seeds: NumPy default-generator seeds 0 (control-cell subsampling, E1/E3/E7), 42 (Tabula Sapiens stratified sampling), and {1,2} (E6 random source-feature draws). All pipeline code is deterministic given these seeds modulo MPS nondeterminism in a small number of matmul reductions.

## 3 Results

### 3.1 Dense, predominantly inhibitory computational graphs

**Figure 2:**
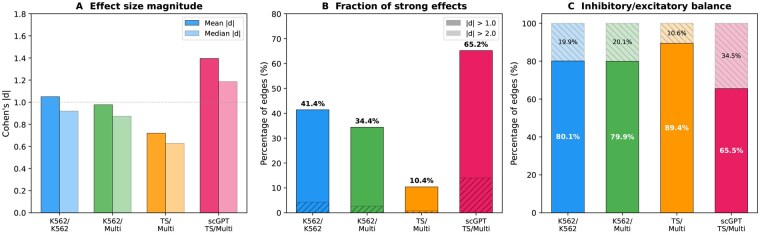
Effect sizes across conditions. (A) Mean and median |d| per condition. (B) Fraction of edges with |d|>1. (C) Sign distribution: inhibitory (*d* < 0) dominates in all four conditions.

Across four conditions we recover 96 892 ablation-derived edges from 80 191 forward passes. The K562/K562 condition (Geneformer, 18 layers) alone contains 52 116 edges connecting 120 source features to 26 338 unique targets (Table 1), corresponding to 31.9% target-feature coverage of the ∼82 944 total features across layers. The 68.1% of target features that receive no significant edge from any of our 120 source features are a mixture of (i) genuinely inactive features on the K562 control corpus (zero or near-zero activation frequency), (ii) features whose activation is driven by upstream features *outside* our 120 annotation-selected source set, and (iii) features whose sensitivity to any of our source features is real but below the |d|>0.5 significance threshold. A saturated tracing pass (all features at all layers as sources) is computationally out of scope here but is a natural extension. The K562/K562 graph has a mean out-degree of 434 unique downstream targets per source feature (52 116 edges across 120 sources). Per-source-feature reach, counted separately at each downstream layer (so the same (s,t) pair at multiple downstream layers contributes multiple entries), falls monotonically with source-layer depth: 2459 layer-entries per L0 feature on average, 1389 at L5, 1033 at L11, 615 at L15 (Table 11, available as supplementary data at *Bioinformatics* online), consistent with attenuation of ablation effects as the propagation distance shrinks. Effect sizes are substantial: mean |d|=1.05, median |d|=0.92, with 41.4% exceeding |d|>1.0 and 4.3% exceeding |d|>2.0 (Fig. 2A).

**Table 1:** Aggregate circuit statistics across four conditions.

Metric	K562/K562	K562/Multi	TS/Multi	TS/Multi
	(GF)	(GF)	(GF)	(scGPT)
Source features	120	90	90	90
Total edges	52 116	8298	5098	31 380
Target features	26 338	4171	2962	1960
Mean |d|	1.05	0.98	0.72	1.40
|d|>1.0 (%)	41.4	34.4	10.4	65.2
Inhibitory (%)	80.1	79.9	89.4	65.5
Shared ontology (%)	52.4	68.5	68.1	50.9

Abbreviations: GF, Geneformer V2-316M; K562/K562: K562 cells with K562-only SAEs; K562/Multi: K562 cells with multitissue SAEs; scGPT, scGPT whole-human; TS/Multi: Tabula Sapiens cells with multitissue SAEs.

A striking property is inhibitory dominance: 80.1% of edges have d<0, meaning that ablating the source feature *reduces* downstream target activation (Fig. 2C). This implies that features predominantly encode *necessary* information—removing a feature causes downstream features that depend on it to lose activation—rather than redundant information (in which removal would free capacity and increase other features). The 20% excitatory fraction reflects disinhibition: ablating some features releases downstream features from suppression. Inhibitory fraction is stable across model (Geneformer, scGPT), cell-type input (K562, Tabula Sapiens), and SAE training data (65.5%–89.4%), identifying it as a model-internal architectural feature rather than a selection artefact. Under binomial sign-randomization, every condition’s inhibitory fraction yields $P<10^{-300}$.

#### 3.1.1 Hub features

Within K562/K562, the top out-degree hubs (most downstream targets) are L0 features annotated with nervous system development (GO: 0007399; 850 targets), golgi organization (GO: 0007030; 825 targets), and RNA methylation (GO: 0001510; 783 targets). Top in-degree hubs (most upstream influence) concentrate in late layers: L16 feature 2818 receives causal input from 93 of 120 source features, and L16 features generally achieve in-degrees of 88–93, indicating that layer 16 serves as a convergent integration layer before the final output layer. In multitissue conditions the hub identity shifts: histone modification (2146 targets), RNA processing (1273), and DNA damage response (562) dominate. Different SAE training data thus drives features to different biological specializations while leaving inhibitory dominance and mean effect size essentially unchanged.

### 3.2 Biological coherence and the annotation-selection caveat

Across the four conditions, 50.9%–68.5% of edges with annotated source and target share at least one GO BP/KEGG/Reactome/STRING/TRRUST term. A configuration-preserving permutation null (500 perms, shuffling term sets within each SAE layer) places the chance rate at 8.5%–23.4% depending on condition, giving fold-enrichments of 2.9–6.2× and empirical P<.002 for every condition (Table 2). However, the 52.4% figure is inflated by the annotation-quality feature selection. Retracing with 20 randomly sampled source features (two independent seeds) yields shared-ontology rates of 21.5% and 26.3%—still 2.5–3.1× above null, but markedly below the annotation-selected value. Edge count, target-feature coverage, mean |d|, and inhibitory dominance are *unchanged* between selection modes, indicating these are circuit-level properties rather than selection effects.

**Table 2:** Permutation baselines for shared ontology.

Condition	Observed (%)	Null mean ± SD (%)	Fold (*P*)
K562/K562 GF (annot.)	52.4	8.5±0.9	6.2 (<.002)
0(random s1)	21.5	≈8.5	2.5
(random s2)	26.3	≈8.5	3.1
K562/Multi GF	68.5	23.4±2.0	2.9 (<.002)
TS/Multi GF	68.1	23.4±2.2	2.9 (<.002)
scGPT TS/Multi	50.9	12.3±1.3	4.1 (<.002)

Observed versus configuration-preserving null (500 perms). “Random-feature” rows are 20 randomly sampled source features at L0.

### 3.3 Circuit magnitude is not reducible to marginal coexpression

A key alternative hypothesis (raised explicitly by our reviewers) is that our ablation graph merely reorganizes marginal gene–gene coexpression already present in the raw data—in which case the circuit would be a convoluted restatement of what a simple correlation matrix already contains. We test this directly. For 31 176 K562/K562 edges whose source and target driver-gene sets are both in the Replogle HVG vocabulary, we compute the maximum absolute Pearson correlation over (s,t)∈sources×targets on 500 K562 nontargeting cells, giving one *marginal coexpression score* per edge. Regressing circuit |d| on this score yields Pearson R=0.098 (Spearman ρ=0.133, $P=8\times 10^{-124}$; $R^{2}=0.0095$): *less than* 1% of the variance in circuit |d| is explained by marginal correlation. The statistical significance follows from n=31 176, not a meaningful effect size—the Pearson correlation coefficient itself is small by any standard. Circuit edges therefore encode structure that bivariate gene–gene correlation on the same cells does not.

A related question is whether circuit edges exhibit *directional asymmetry* inaccessible to symmetric correlation—e.g. whether the model treats A→B and B→A differently. Our strictly forward ablation design (source layer always earlier than target layer) prevents a within-layer asymmetry test on the current circuit graph; this is a limitation we flag for future work involving same-layer cross-direction formulations or recurrent circuit tracing.

### 3.4 Causal effects persist across network depth

A fundamental question is how far ablation effects propagate. For K562/K562 Geneformer, L0 features produce ∼2459 significant edges on average (ranging to targets at all 17 downstream layers), maintaining more than 200 significant edges for five downstream layers before decaying (see Table 11, available as supplementary data at *Bioinformatics* online, for per-source-layer breakdown); L15 features show an *increase* in effect at L17, suggesting late-layer consolidation of information. For scGPT TS/Multi, L0 effects plateau through ∼L6 before declining, with L4 features the most broadly connected (contrasting Geneformer, where L0 dominates). The persistence profile is model-specific: Geneformer propagates early layer features throughout depth, while scGPT concentrates influence in midlayers. Coactivation PMI edges overlap 91%–95% with ablation-derived targets at matched layer pairs, cross-validating both methods; ablation uniquely provides directionality, sign, and magnitude.

### 3.5 Interpretable circuit examples

Manual examination of the strongest annotated edges yielded canonical multistep biology encoded as directed circuits. The multitissue DDR cascade (source feature $f_{1372}$ at L0 labeled “DNA Damage Response”) drives $f_{4410}$ at L5 (“DNA Unwinding Involved In DNA Replication,” d=−5.98), $f_{2576}$ at L5 (“G1/S Transition,” d=−3.2), and $f_{3309}$ at L11 (“G2/M Transition,” d=−2.1), mirroring the canonical damage-sensing → checkpoint → arrest pathway (Jackson and Bartek 2009, Ciccia and Elledge 2010); the G1/S and G2/M features correspond to the CDK-regulated transitions central to the canonical cell cycle (Malumbres and Barbacid 2009) and the spindle-assembly checkpoint (Musacchio and Salmon 2007). A nervous-system-development hub at L0 ($f_{146}$) drives seven layer-1-to-13 targets spanning proteostasis (Ross and Poirier 2004, Labbadia and Morimoto 2015), vesicle transport, and immune signaling—each sharing ≥128 ontology terms with the source. An scGPT protein-catabolism hub at L0 ($f_{3101}$) drives the strongest individual edges in either model (d=−8.19 to chromatin organization, d=−6.10 to DNA metabolism), consistent with the tight coupling between protein-turnover machinery and transcriptional regulation observed during the unfolded-protein response (Walter and Ron 2011, Hetz 2012) and under nucleolar stress (Boulon *et al.* 2010). We report 20+ similar circuits in Supplementary Material, available as supplementary data at *Bioinformatics* online, including examples where the hub biology recapitulates MAP-kinase signaling (Dhillon *et al.* 2007), Wnt/β-catenin signaling (Nusse and Clevers 2017), cholesterol transport (Simons and Ikonen 2000), and mitochondrial electron transport (Chandel 2021).

### 3.6 Input size normalized cross-model comparison

**Figure 3:**
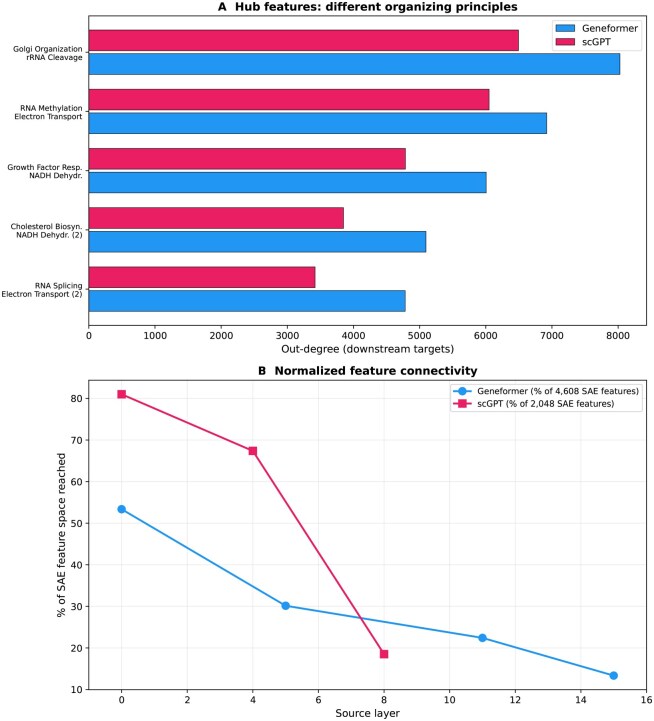
Cross-model comparison. (A) Different hub biology: Geneformer organizes around Golgi/RNA processing; scGPT around rRNA cleavage/mitochondrial electron transport. (B) Normalized connectivity (fraction of SAE feature space reached per source feature). Denominators are SAE dictionary sizes (4608 Geneformer, 2048 scGPT), not model context windows.

scGPT edges are stronger in raw magnitude (mean |d|=1.40 versus 1.05), but scGPT uses 2048 SAE features and an input length of 1200 padded positions, while Geneformer uses 4608 features and a context window of 4096 tokens. Each feature carries a larger representational share in scGPT. Under *feature-share normalization* $d_{\text{norm}}=d\times n_{\text{features}}/4608$, the scGPT mean is 0.62, while Geneformer is unchanged at 1.05: *Geneformer is stronger per-feature*. For 33 301 gene pairs common to both models’ circuits, the raw scGPT/Geneformer |d| ratio is 1.43 but the feature-share normalized ratio is 0.64, and sign agreement between models is 66.3% with cross-model Pearson r(d)=0.10. The “scGPT stronger effects” reading based on raw |d| is therefore an artifact of the smaller SAE dictionary, not a true per-feature magnitude difference. Figure 3B visualizes the underlying connectivity asymmetry.

### 3.7 Cross-model consensus and disease centrality

Despite architectural divergence (transformer depth, hidden dimension, tokenization scheme, training data), Geneformer and scGPT independently recover 1142 identical ordered domain pairs (GO BP source → target)—a 10.6× enrichment over a 1000-permutation null (expected 107.3; empirical P<0.001), and visually reflected in the cross-model hub contrast of Fig. 3. Of these, 303 are high-confidence pairs in which both models show mean |d|>1. Example consensus pairs include DNA damage response → DNA-templated DNA replication (both models, all four conditions; mean $|d{|}_{\mathrm{Geneformer}}=3.9$, $|d{|}_{\mathrm{scGPT}}=4.2$), NADH dehydrogenase complex assembly → mitochondrial respiratory chain complex assembly, and cholesterol biosynthetic process → sterol biosynthetic process.

Disease-associated domains—defined by keyword matching over 11 categories (DNA damage/repair, cell cycle, apoptosis, immune response, oncogenic signaling, protein quality control, angiogenesis, metastasis, metabolism) plus TRRUST transcription factors—are substantially more central in the graph: median 14 circuit edges versus median 3 for nondisease domains (Mann–Whitney $P=1.2\times 10^{-11}$) and 3.59× more likely to be cross-model consensus pairs (P<.001, Fisher’s exact). This does not mean all disease-relevant gene sets are equally enriched—the 11 categories are intentionally broad, and most circuit domains match at least one. The informative finding concerns *relative centrality* and *cross-model conservation*: the biology most relevant to human disease is also the biology most robustly encoded across architectures, and sits at the hub of the circuit graph rather than its periphery.

### 3.8 Matched-cell-type regulatory coherence

The paper’s initial TRRUST (merged-TF-network) check reported only 1.12× enrichment of circuit gene-pair predictions in known regulatory edges. A matched-cell-type ChIP-seq prior is much more appropriate. Against the ENCODE 5-cell-line TF–target edge set (K562 the largest contributor), K562/K562 circuit predictions are enriched 2.06× (Fisher OR 5.84, P≈0); all four conditions show 1.41–2.06× enrichment (all p≈0). Restricting to the 100 TFs with any K562 ChIP-seq experiment yields 1.82× enrichment (OR 6.20) for K562/K562; OR is stable between pooled and K562-restricted sets, arguing the signal is not a pooling artefact.

### 3.9 Direct versus indirect target partition

R1 noted that Perturb-seq responses conflate direct and indirect targets, and that the Geneformer paper reported stronger *in silico* effects on ChIP-seq direct targets. We therefore partitioned every validation gene pair (s,t) into *direct* (when (s,t) is an edge in the ENCODE K562-restricted set) or *indirect*. On K562, direct-pair excess over sign-bias null is +0.89 percentage points (n=3545; 56.05% versus 55.16%) versus +0.03 pp indirect (n=781 542)—a ∼30× ratio. On RPE1, direct excess is +1.03 pp (60.27% versus 59.24%; n=5009) versus +0.35 pp indirect (n=811,401)—a 3× ratio. Spearman $\rho (|d|,|{\;\text{log}\;}_{2}\text{FC}|)$ is weakly *negative* on direct pairs (−0.12 K562, −0.17 RPE1; both $P<10^{-13}$): stronger circuit edges target genes with smaller measured perturbation response, plausibly reflecting buffering of core regulatory targets.

### 3.10 Systematic knowledge extraction across 96 892 edges

To move beyond selected circuit vignettes, we annotated every edge across all four conditions with source and target GO BP domain labels, yielding 37 088 edges (38.3%) where both endpoints are annotated. Of the resulting 16 067 unique ordered domain pairs, 29 864 annotated edges (80.5%) connect domain pairs *not* linked in a reference “known biology” graph built from GO/KEGG/Reactome gene-overlap edges (gene-overlap ≥3, 14 021 reference links). These 80.5% of edges are candidate novel domain-level relationships: domain pairs the model has decided to connect that are not obvious from curated pathway databases. We do not claim biological validity for each—many are likely co-regulation artefacts—but the set is a structured hypothesis space for targeted experimental follow-up, especially when restricted to the 1142cross-model consensus pairs. Extracting per-edge gene-level predictions (top 10 driver genes per feature, rank-weighted, filtered for ≥2 independent edges or |d|>2) yields 975369 gene-pair predictions; 0.15% match STRING/TRRUST, 1.14% share ≥2 GO BP terms; the remaining 98.7% are candidate, not-yet-documented relationships.

### 3.11 CRISPRi validation with sign-bias correction

A methodological point: directional-accuracy numbers from ablation-versus-perturbation comparisons are substantially biased whenever predictions and observations have skewed sign marginals. Circuit *d* values are ∼85% negative (a direct consequence of the 65%–89% inhibitory dominance); measured CRISPRi ${\;\text{log}\;}_{2}\text{FC}$ is 53%–59% negative (because many knockdowns reduce many downstream genes through indirect cell-state effects, not just the intended direct targets). Under *independence* of these marginals, sign-agreement would already be 52%–56% purely from bias—so a reported “directional accuracy of 56.4%” reflects essentially zero model-relevant signal once the bias is accounted for. We therefore report accuracy alongside the sign-bias null Null=P(pred<0)P(obs<0)+P(pred>0)P(obs>0) and focus on *excess over this null*. This correction is not standard in foundation-model interpretability papers but substantially changes how results are interpreted; we recommend it as a routine check whenever predicted and observed sign distributions are both known to be skewed. On K562 Replogle (aggregated as |d|-weighted mean per (s,t) pair): directional accuracy 52.08% versus bias null 52.06%, excess +0.03 pp (n=785 087 pairs, 323 source genes; Spearman ρ=−0.005). On RPE1: 56.40% versus 56.05%, excess +0.35 pp (n=816 410, 339 source genes; ρ=+0.007). Effect-magnitude correlations are essentially zero in both. A pool-level guide-efficacy filter (${\;\text{log}\;}_{2}\text{FC}<-0.5$, P<.05) is passed by 1309/1309 K562 and 1446/1446 RPE1 targets and does not change the numbers. Table 3 summarizes all seven validation rows (K562 all/RPE1 all/Shifrut plus direct/indirect splits for K562 and RPE1).

**Table 3:** CRISPRi validation summary with sign-bias correction.

Screen	Partition	*n* pairs	Dir. acc. (%)	Excess
K562 Replogle (all)	All	785 087	52.08	+0.03 pp
RPE1 Replogle (all)	All	816 410	56.40	+0.35 pp
Shifrut primary T-cell	All	1557	50.35	−0.03 pp
K562 Replogle	Direct	3545	56.05	+0.89 pp
K562 Replogle	Indirect	781 542	52.07	+0.03 pp
RPE1 Replogle	Direct	5009	60.27	+1.03 pp
RPE1 Replogle	Indirect	811 401	56.38	+0.35 pp

Dir. acc. is directional sign agreement between circuit *d* and measured ${\;\text{log}\;}_{2}\text{FC}$. Sign-bias null is P(pred<0)P(obs<0)+P(pred>0)P(obs>0). Excess is the above-null signal. Direct/indirect refers to the ENCODE K562 ChIP-seq partition.

#### 3.11.1 Truly nonimmortalized primary-cell control

Shifrut *et al.*’s (2018) primary human CD8${\;}^{+}$ T-cell CRISPR-KO (52 236 cells, 20 immune-checkpoint targets, 30 683 controls) is nonimmortalized and nonmalignant. Two caveats constrain interpretation: (i) CRISPR-KO preserves transcript (median self-${\;\text{log}\;}_{2}\text{FC}=-0.03$; R1’s CRISPRi-scale filter retains zero guides, and a KO-scale relaxation is needed); (ii) only 1/20 Shifrut targets overlaps the K562 L0 annotation-selected source driver set, because annotation-selected features at L0 encode housekeeping/DNA-damage/cell-cycle biology rather than specialized immune checkpoint regulation. Result: n=1557 pairs, dir. acc. 50.35% versus bias null 50.68% (excess −0.03 pp); Spearman ρ=−0.02. We report this null result transparently: K562-trained annotation-selected circuits do not transfer to primary-T-cell immune-checkpoint biology, consistent with the feature-selection bias documented above. A properly matched test requires T-cell-targeted source-feature selection or a primary-cell CRISPRi screen with immune-checkpoint coverage.

### 3.12 Stability: sample size and cell-type decomposition

Retracing L0 at N∈{50,100,200} with the same 20 annotation-selected features: Cohen’s *d* values at shared edges are highly stable (Pearson r=0.973 for N=50 versus N=200, r=0.989 for N=100 versus N=200; 100% sign agreement on every pair). Edge-identity stability grows monotonically with *N*: edge Jaccard is 0.44 (N=50 versus N=200), 0.57 (N=50 versus N=100), and 0.64 (N=100 versus N=200); 78.9% of N=200 edges are recovered at N=100; 63.5% at N=50. N=200 is therefore the conservative filter: edges near the |d|>0.5 threshold fluctuate with sample size and are more reliably captured at higher *N*, while the effect-size *values* for an edge that survives the threshold at any *N* are near-identical across *N*. The paper’s quantitative headline numbers are all reported at N=200 and survive this stability check.

Per-cell-type Tabula Sapiens retracing (N=50 from a single immune type at a time, multitissue SAEs at layers {0,5,11,17}): each per-type graph contains ∼2370 edges (B cell 2402; CD4${\;}^{+}$ T 2359; macrophage 2364; stratified 2375). Pairwise Jaccard between immune types is 0.18–0.26; Pearson r(d) on shared edges 0.90–0.96; sign agreement >99.8%. The stratified 200-cell/88-type graph overlaps each single-type graph at 17%–20% Jaccard—consistent with its being a genuine mixture that no single cell type dominates. All pairwise values are summarized in Table 4.

**Table 4:** Stability of the circuit graph across sample size and cell-type decomposition.

Comparison (L0)	Jaccard	Pearson r(d)	Sign agree. (%)
Sample size stability (K562/K562)			
N=50 versus N=100	0.57	0.985	100.0
N=50 versus N=200	0.44	0.973	100.0
N=100 versus N=200	0.64	0.989	100.0
Per-immune cell type (TS/Multi)			
B cell ↔ CD4${}^{+}$T	0.26	0.949	100.0
B cell ↔ macrophage	0.22	0.944	100.0
CD4${}^{+}$T ↔ macrophage	0.18	0.905	99.9
B cell ↔ stratified 88-type	0.18	0.941	100.0
CD4${}^{+}$T ↔ stratified 88-type	0.18	0.940	100.0
Macrophage ↔ stratified 88-type	0.20	0.956	100.0

Pairwise comparisons on the L0 subgraph (20 annotation-selected features for sample size row; ∼2370 edges per per-cell-type row).

Two interpretive notes. First, per-type edge Jaccards of 0.18–0.26 are substantially lower than the 0.44–0.64 seen in the sample size stability comparison at matched feature sets. This difference reflects genuine cell-type specificity: the edges a B-cell circuit uses are not the same edges a macrophage circuit uses, even with identical source-feature selection. Second, Pearson r(d) on shared edges stays high (≥0.90) across cell types, so when two cell types *do* recruit the same edge, its effect size is preserved. This separates “which edges are active” (cell-type-specific) from “how strong each active edge is” (cell-type-invariant).

## 4 Discussion

### 4.1 What we learned

Both Geneformer and scGPT compute with dense, inhibitory, partially redundant circuits. Inhibitory dominance (65%–89%) is an invariant across architecture, cell type, and SAE training data: each feature encodes information that downstream features depend on, rather than redundant information. Shared-ontology rates of 50%–68% on annotation-selected features, attenuating to 22%–26% on random features, are both well above chance (2.5–6.2×) but quantitatively weaker than a pure biological-encoding interpretation would predict. Circuit edge magnitude is not reducible to marginal co-expression ($R^{2}<0.01$). Cross-model consensus at 10.6× enrichment identifies 1142 architecture-invariant domain pairs that are strong candidates for mechanisms the models have genuinely learned.

### 4.2 What the models *do not* do

They do not reliably predict CRISPRi perturbation responses. After sign-bias correction, excess over baseline is +0.03 pp on K562 and +0.35 pp on RPE1; effect-magnitude Spearman correlations are essentially zero. Predictions do not transfer to a primary human T-cell CRISPR-KO screen either, not just because of KO versus CRISPRi differences in transcript behavior but because the K562-trained annotation-selected source features barely overlap with T-cell checkpoint biology at the gene level (only 1 of Shifrut’s 20 immune-checkpoint targets is in the K562 circuit source driver set). This is less a failure of the tracing method than a corollary of the feature-selection bias: features scored by ontology-enrichment quality concentrate in housekeeping biology (cell cycle, DNA damage, RNA processing, mitochondrial respiration) that is well annotated and well represented in the training corpus, at the cost of specialized regulatory biology that is less densely annotated. Circuits traced from a source pool biased toward housekeeping therefore validate weakly against a perturbation screen biased toward checkpoint regulation.

### 4.3 The direct-target nuance

Partitioning validation pairs by ChIP-seq support reveals that direct ChIP-seq-supported target pairs show 10–30× higher excess over bias than indirect pairs in both K562 and RPE1. The models capture a weak but detectable direct regulatory signal that is invisible on Perturb-seq-all-genes benchmarks, matching the Geneformer paper’s original observation at higher resolution.

### 4.4 Limitations

(i) Annotation-selected features inflate coherence numbers by ∼2.5×; all headline rates should be read alongside the random-feature baseline. (ii) Our CRISPRi validation remains K562-centric despite the RPE1 addition because RPE1 is hTERT-immortalized; the Shifrut primary-T-cell test is a principled null due to the checkpoint-biology versus annotation-selected-feature mismatch. (iii) The strictly forward ablation design (source layer earlier than target layer) prevents direct tests of directional asymmetry within layer. (iv) Dynamic cellular processes such as differentiation trajectories, which would naturally be studied using diffusion pseudotime (Haghverdi *et al.* 2016) or related lineage-ordering methods, are here captured only statically via SAE feature activations on the Tabula Sapiens atlas; a fully dynamic circuit analysis integrating pseudotime ordering with ablation-derived edges is future work.

### 4.5 Relation to prior work

Mechanistic interpretability has primarily been developed in language models (Wang *et al.* 2023, Bereska and Gavves 2024, Marks *et al.* 2025); our work extends the paradigm to biological foundation models at scale. The Geneformer paper (Theodoris *et al.* 2023) reported that *in silico* deletion effects are stronger for ChIP-seq direct targets than for Perturb-seq-wide targets. Our direct versus indirect partition corroborates this observation quantitatively (10–30× excess over bias ratio) and generalizes it from a handful of case-study TFs to a statistically grounded graph-wide test. Prior SAE atlas work for Geneformer and scGPT established *what* features exist; the present work establishes *how* features causally relate. The cross-model consensus set of 1142 domain pairs is a testable prediction list for future wet-lab validation.

### 4.6 Implications for scFM interpretability

Three findings matter for the field. First, inhibitory dominance (65%–89%) means current scFMs compute with circuits structured around *necessity* rather than redundancy: ablation tools will therefore see mostly large, negative effects—the apparent abundance of edges is partly an accounting consequence which our sign-bias correction explicitly accounts for. Second, annotation-selected feature subsets systematically overrepresent interpretable biology; any interpretation paper using such a subset should separately report the random-feature baseline. Third, the gap between high model-internal causal reproducibility (r≥0.97) and low biological causal fidelity (+0.03/+0.35 pp excess) indicates that improvements in biological interpretability will come from changes upstream—in scFM training objectives (e.g. perturbation-conditional supervision using Perturb-seq Dixit *et al.* 2016) or in feature selection (filtering for driver-gene relevance). Our circuit graph is consistent with scFMs having learned the *descriptive* structure of transcription in substantial detail but only weakly encoding the *interventional* structure.

### 4.7 Why causal tracing beats correlation-based interpretability

Coactivation statistics (pointwise mutual information) identify groups of features that fire together. As our PMI-versus-ablation comparison shows, most (91%–95%) of a source feature’s ablation targets also appear as high-PMI partners, so either method recovers a similar support set. What they return beyond that support is different: PMI yields undirected, unsigned edges between simultaneously active features, while ablation assigns direction (source → target is ordered by network depth), sign (whether removing the source raises or lowers the target), and magnitude (Cohen’s *d*). The combination lets us ask specifically mechanistic questions—e.g. “what does an upstream DNA-damage feature cause to happen at a late-layer cell-cycle feature?”—which co-activation alone cannot.

### 4.8 Outlook

Making circuit predictions clinically actionable will require (i) feature-selection schemes that explicitly target cell-type-specific regulation (e.g. filtering features whose driver genes intersect a cell-type-relevant gene set), (ii) primary-cell CRISPRi screens that cover the biology the circuits actually encode, or (iii) retracing circuits using a source pool expanded beyond the annotation-qualified top-*k*—e.g. all features whose driver-gene sets overlap a chosen disease gene list. The methodological tools released here—sign-bias-corrected validation, direct versus indirect target partitioning, configuration-preserving permutation nulls, feature-share effect-size normalization, and random-source-feature baselines—are not specific to the two models we studied and should apply to any future biological FM with a trained SAE atlas. The cross-model consensus graph of 1142 domain pairs, released alongside the code, is a testable prediction list for targeted Perturb-seq follow-up.

## Supplementary Material

btag379_Supplementary_Data

## Data Availability

https://github.com/Biodyn-AI/bio-sae-circuits (Python). Archival DOI: 10.5281/zenodo.19633166 (Zenodo).
