# Supplementary material for: Causal circuit tracing reveals distinct computational architectures in single-cell foundation models: inhibitory dominance, biological coherence, and cross-model convergence
Source: Bioinformatics. 2026 Jun 15;42(7):btag379. doi: 10.1093/bioinformatics/btag379 (PMC13354606; doi:10.1093/bioinformatics/btag379)
Supplement: btag379_Supplementary_Data [file btag379_supplementary_data.pdf]

Supplementary Material

Causal circuit tracing reveals distinct computational architectures in single-cell foundation models: inhibitory dominance, biological coherence, and cross-model convergence

Ihor Kendiukhov

Contents

S1 Expanded permutation baselines **2**

S2 Threshold sensitivity analysis **2**

S3 Complete CRISPRi validation details **3**
S3.1 Pseudobulking protocol 3
S3.2 Efficacy filter outcomes 4
S3.3 Pair-aggregation protocol 4
S3.4 Sign-bias null formula 4

S4 Stability: detailed breakdowns **4**

S5 Expanded interpretable circuit examples **5**
S5.1 DDR → cell cycle cascade (multi-tissue Geneformer) 5
S5.2 Neurodevelopment–proteostasis hub (Geneformer K562/K562) 5
S5.3 Protein-catabolism hub (scGPT) 5

S6 Partial correlation between circuit $|d|$ and marginal co-expression (E7) **6**
S6.1 Protocol 6
S6.2 Result 6

S7 ENCODE ChIP-seq prior construction **6**
S7.1 5-cell-line edge set 6
S7.2 K562-restricted subset 6

S8 Complete systematic knowledge extraction **7**
S8.1 Disease circuit mapping 7

S9 Random-feature control **8**

S10 Input-size normalisation detail (E5) **8**

S11 Per-source-layer circuit statistics **9**

S1 Expanded permutation baselines

Table S1 reports the complete shared-ontology permutation nulls (500 permutations per condition) and inhibitory-dominance binomial tests, including confidence intervals.

Table S1: **Permutation baselines and sign-randomisation null (complete).**

Condition	Obs. shared	Null mean ± SD	Fold	Emp. p	Inhib. binom. p
K562/K562 GF (annot.)	52.4%	$8.5 \pm 0.9\%$	6.2	< 0.002	$< 10^{-300}$
(random seed 1)	21.5%	$\approx$8.5%	2.5	< 0.002	$< 10^{-300}$
(random seed 2)	26.3%	$\approx$8.5%	3.1	< 0.002	$< 10^{-300}$
K562/Multi GF	68.5%	$23.4 \pm 2.0\%$	2.9	< 0.002	$< 10^{-300}$
TS/Multi GF	68.1%	$23.4 \pm 2.2\%$	2.9	< 0.002	$< 10^{-300}$
scGPT TS/Multi	50.9%	$12.3 \pm 1.3\%$	4.1	< 0.002	$< 10^{-300}$

Cross-model consensus permutation. For the 1,142 architecture-invariant domain pairs reported in main-text §3.7, the 1,000-permutation null (shuffling domain labels within model) gives expected 107.3 pairs and observed 1,142 ($p < 0.001$, all 1,000 permutations < observed). Of these, 303 pass a high-confidence threshold (both models mean $|d| > 1.0$); the high-confidence-enrichment null permutation yields expected 14.7 vs. observed 303 (20.6×, $p < 0.001$).

S2 Threshold sensitivity analysis

We swept the $|d|$ cutoff over $\{0.1, 0.2, 0.3, 0.5, 0.7, 1.0\}$ at fixed consistency > 0.7. Because circuit tracing was initially filtered at $|d| > 0.5$, values below 0.5 subset the existing graph rather than adding lower-magnitude edges (this requires re-running the raw ablation pipeline, which is deferred to future work). We therefore report the behaviour of headline metrics at $\{0.5, 0.7, 1.0\}$ (Table S2). All metrics are stable within 6 percentage points across this range for every condition.

Table S2: **Threshold sensitivity sweep.** $|d| \geq t$ for each condition. Inhib. = inhibitory edge fraction. Shared = ontology-set-intersection shared-ontology fraction.

Condition	Metric	t=0.5	t=0.7	t=1.0
K562/K562 GF	N edges	52,116	41,390	21,573
	Mean $\lvert d \rvert$	1.05	1.16	1.46
	Inhib. (%)	80.1	80.3	80.3
	Shared (%)	52.4	53.4	58.0
K562/Multi GF	N edges	8,298	6,398	2,852
	Mean $\lvert d \rvert$	0.98	1.09	1.39
	Inhib. (%)	79.9	81.2	83.5
	Shared (%)	68.5	69.8	72.1
TS/Multi GF	N edges	5,098	1,783	530
	Mean $\lvert d \rvert$	0.72	0.98	1.37
	Inhib. (%)	89.4	87.3	90.8
	Shared (%)	68.1	74.3	76.5
scGPT TS/Multi	N edges	31,380	29,514	20,452
	Mean $\lvert d \rvert$	1.40	1.44	1.71
	Inhib. (%)	65.5	64.5	63.1
	Shared (%)	50.9	51.9	55.2

FDR-controlled threshold. Under a Welch t-test null at n=200 with Benjamini–Hochberg correction over ~550,000 hypothesis tests, the critical $|d|$ at FDR < 0.05 is 0.39 (parametric upper bound). The paper's $|d| > 0.5$ threshold is therefore more conservative than statistical FDR control; every reported edge is FDR-significant.

Consistency-threshold sensitivity. The consistency statistic, $c = \frac{\#\{\text{cells with sign}(\Delta_i)=\text{sign}(\bar{\Delta})\}}{N}$, removes edges whose sign is carried by only a small minority of cells. Varying the threshold c_* at fixed $|d| > 0.5$ on K562/K562:

c_*	N edges	Mean $\lvert d \rvert$	Inhibitory%	Shared-ontology%
0.6	54,891	1.04	79.8	52.1
0.7 (paper)	52,116	1.05	80.1	52.4
0.8	48,904	1.07	80.6	53.0

Headline counts and rates are stable within ±8% across this range; the paper's $c_* = 0.7$ sits mid-range and reflects the empirical inflection where noise edges begin to dominate.

S3 Complete CRISPRi validation details

S3.1 Pseudobulking protocol

For each cell line (K562 and RPE1), we identified cells bearing guides for each targeted gene and pooled them. For each target gene g, we computed the pseudobulk expression mean $\bar{x}_g^{\text{pert}} = \log_2\left(\frac{1}{N}\sum_{i\in\text{pert}(g)} x_i + 10^{-3}\right)$ and similarly for non-targeting controls $\bar{x}^{\text{NT}}$; log-fold-change per gene

j is $\bar{x}_{g,j}^{\text{pert}} - \bar{x}_j^{\text{NT}}$. Cells per target pool are sub-sampled to 2,000 if exceeding this number (controls sub-sampled to 5,000) for computational efficiency. Guide efficacy is assessed by Welch's t-test on the targeted-gene expression distribution between pert and NT cells.

S3.2 Efficacy filter outcomes

Table S3: **Guide-efficacy filter pass rates per screen.**

Screen	Filter threshold	N targets	N passing
Replogle K562	CRISPRi-scale ($\log_2$ FC < -0.5, $p < 0.05$)	1,309	1,309
Replogle RPE1	CRISPRi-scale	1,446	1,446
Shifrut T cells	CRISPRi-scale	20	0
Shifrut T cells	KO-scale ($\log_2$ FC < -0.04, $p < 0.05$)	20	9

S3.3 Pair-aggregation protocol

For each circuit edge $e = (f_{\text{src}}, f_{\text{tgt}})$ with source-driver gene set S_e and target-driver gene set T_e, we enumerate Cartesian products $S_e \times T_e$ (capping at the top-15 driver genes per side) and exclude self-edges. Multiple edges covering the same gene pair (s, t) are aggregated by $|d|$-weighted mean. Only gene pairs where s is a perturbation target and t is in the var vocabulary enter the validation set.

S3.4 Sign-bias null formula

Let $p_- = P(\text{pred} < 0)$ and $q_- = P(\text{obs} < 0)$ be the marginal negative-sign fractions over evaluated pairs. Under independence, the expected sign-agreement is

$$\text{null} = p_- q_- + (1 - p_-)(1 - q_-).$$

The reported "excess-over-bias" equals (observed directional accuracy) − (this null).

S4 Stability: detailed breakdowns

Table S4: **Bootstrap stability: full table.** Edges at $|d| > 0.5$ on L0 sub-graph with same 20 annotation-selected features across $N \in \{50, 100, 200\}$.

Comparison	Edges N_1	Edges N_2	Intersection	Edge Jaccard	Pearson $r(d)$
N=50 vs. N=100	16,469	15,964	11,711	0.565	0.985
N=50 vs. N=200	16,469	15,504	9,839	0.445	0.973
N=100 vs. N=200	15,964	15,504	12,238	0.636	0.989

Per-source-feature recall. Of the 15,504 edges at N=200 (within the 20-feature common set), 12,238 (78.9%) are recovered at N=100 and 9,839 (63.5%) at N=50. Edges with $|d| > 1$ at N=200 are recovered at N=50 at 89.3% rate (vs. 63.5% for the full set), confirming that edge persistence correlates with effect-size magnitude.

Table S5: **Per-cell-type circuit stability (Tabula Sapiens, multi-tissue SAEs).**

Comparison	n_1	n_2	Intersection	Jaccard	Pearson $r(d)$
B cell ↔ CD4$^+$T	2402	2359	992	0.263	0.949
B cell ↔ Macrophage	2402	2364	864	0.221	0.944
CD4$^+$T ↔ Macrophage	2359	2364	726	0.182	0.905
B cell ↔ Stratified(88-type)	2402	2375	742	0.184	0.941
CD4$^+$T ↔ Stratified	2359	2375	704	0.175	0.940
Macrophage ↔ Stratified	2364	2375	793	0.201	0.956

S5 Expanded interpretable circuit examples

S5.1 DDR → cell cycle cascade (multi-tissue Geneformer)

Source feature f_{1372} at L0 labelled "DNA Damage Response (GO:0006974)" (driver genes: MCM6, UNG, MCM5, MCM2, CDC6, CDK2, MCM4, GINS2, E2F1, MCM3, UHRF1) drives a cascade across four layers:

- f_{4410} at L5: "DNA Unwinding Involved In DNA Replication (GO:0006268)" ($d = -5.98$)
- f_{2576} at L5: "G1/S Transition (GO:0000082)" ($d = -3.2$)
- f_{3309} at L11: "G2/M Transition (GO:0000086)" ($d = -2.1$)
- f_{2051} at L17: "DNA Damage Response" recurrence ($d = -2.8$)

All edges are inhibitory, consistent with the canonical checkpoint topology in which DDR activation suppresses replication until damage is repaired.

S5.2 Neurodevelopment–proteostasis hub (Geneformer K562/K562)

Source f_{146} at L0 labelled "Nervous System Development (GO:0007399)" drives 7 targets across layers 1–13 spanning proteostasis (Golgi Vesicle Transport, Modification-Dependent Protein Catabolic Process), cellular transport (Endosome Organization, Intracellular Protein Transport), and immune signalling, with 128–142 shared ontology terms per edge. All edges are inhibitory, with $d \in [-1.66, -0.87]$.

S5.3 Protein-catabolism hub (scGPT)

L0 feature f_{3101} labelled "Maturation Of SSU-rRNA (GO:0030490)" (by annotation, but gene drivers include UBB, PSMB2, PSMD7, indicating UPR/proteostasis overlap) drives the strongest individual edges in either model:

- $d = -8.19$ to Chromatin Organization (L1)
- $d = -6.10$ to DNA Metabolism (L1)
- $d = -3.84$ to Macromolecule Biosynthesis (L5)

S6 Partial correlation between circuit $|d|$ and marginal co-expression (E7)

S6.1 Protocol

For each circuit edge e with source driver gene set S_e (capped at 20 genes) and target driver gene set T_e (capped at 20 genes):

1. Load 500 K562 non-targeting cells from Replogle (sub-sampled).
2. For each gene g in $S_e \cup T_e$, standardise expression to zero mean, unit variance.
3. Compute $\mathrm{corr}(s,t) = \mathbb{E}[z_s z_t]$ for all $s \in S_e$, $t \in T_e$.
4. Edge co-expression score: $\mathrm{coexp}(e) = \max_{s,t} |\mathrm{corr}(s,t)|$.
5. Regress $|d_e|$ on $\mathrm{coexp}(e)$ across all edges.

S6.2 Result

Across 31,176 edges with both driver sets in vocabulary:

- Pearson $R(|d|, \mathrm{coexp}) = 0.098$; $R^2 = 0.0095$
- Spearman $\rho = 0.133$, $p = 8.39 \times 10^{-124}$
- Mean coexp $= 0.69$; mean $|d| = 1.08$

Interpretation. Less than 1% of variance in circuit edge magnitude is explained by marginal co-expression of the driver genes. The Spearman p-value's size reflects $n = 31{,}176$, not effect magnitude ($\rho = 0.13$ is small).

S7 ENCODE ChIP-seq prior construction

S7.1 5-cell-line edge set

The edge set `encode_tf_targets_5celllines_edges.tsv` contains 1,521,659 TF–target edges covering 184 TFs and 22,076 target genes. The universe is $184 \times 22{,}076 = 4{,}061{,}984$ possible pairs with background density 0.375. This set combines ChIP-seq peaks for the five cell lines with the most experiments: K562 (150/690 of ENCODE TFBS clusters), GM12878 (90), HepG2 (77), HeLa-S3 (64), H1-hESC (58).

S7.2 K562-restricted subset

Filtering to only those TFs with at least one K562 ChIP-seq experiment in `wgEncodeRegTfbsClusteredInputsV3.ta` (cell line column equals "K562") retains 100 TFs, 21,973 targets, and 943,160 edges. The corresponding universe is 2,065,462 with density 0.457.

Table S6: **ChIP-seq enrichment summary (full).**

Prior	Condition	Pred. pairs	Overlap	Enrichment	Fisher p
5-line ENCODE	K562/K562 GF	102,584	78,980	2.055×	≈ 0
	K562/Multi GF	27,865	16,650	1.595×	≈ 0
	TS/Multi GF	19,627	11,790	1.604×	≈ 0
	scGPT TS/Multi	35,699	18,900	1.413×	≈ 0
K562-restricted	K562/K562 GF	68,789	57,222	1.822×	≈ 0
	K562/Multi GF	11,962	9,776	1.790×	≈ 0
	TS/Multi GF	8,624	7,129	1.810×	≈ 0
	scGPT TS/Multi	25,072	14,923	1.303×	≈ 0

S8 Complete systematic knowledge extraction

Table S7: **Knowledge extraction across all 96,892 edges.**

Metric	Value	Note
Total edges	96,892	Across 4 conditions
Both endpoints annotated	37,088	38.3% of edges
Unique domain pairs	16,067	1,126 unique domains
Cross-model consensus pairs	1,142	10.65× over null
High-confidence consensus	303	$\lvert d\rvert > 1$ in both models
Novel pairs (all 4 conditions)	87	Top $\lvert d\rvert$ up to 7.21
Candidate gene-pair predict.	975,369	After filter: ≥ 2 edges or $\lvert d\rvert > 2$
STRING/TRRUST match	0.15%	Most predictions are novel
$\geq$2 shared GO BP terms	1.14%	Above-chance structure

S8.1 Disease circuit mapping

Table S8: **Disease-relevant circuit summary.**

Disease category	Domains	Circuit edges	Consensus pairs
DNA damage/repair	52	7,483	178
Cell cycle	44	9,201	221
Apoptosis	19	2,306	48
Immune response	63	4,015	85
Oncogenic signalling	41	2,844	72
Protein quality control	28	3,112	91
Angiogenesis	18	934	22
Metastasis	25	1,558	41
Metabolism	87	7,296	143
TRRUST TFs	152	2,510	158

Centrality. Disease-associated domains are 4.7× more central (median 14 vs. 3 circuit edges; Mann-Whitney $p = 1.2 \times 10^{-11}$) and 3.59× more likely to appear in cross-model consensus ($p < 0.001$,

Fisher's exact).

S9 Random-feature control

Table S9: **Random vs. annotation-selected source features (L0, N=50).**

Metric	Annotation-selected	Random seed 1	Random seed 2
N edges	16,469	16,966	16,594
N target features	13,262	14,086	13,508
Mean $\|d\|$	1.23	1.15	1.14
Median $\|d\|$	0.96	0.93	0.92
Inhibitory fraction (%)	84.7	88.2	88.3
Shared ontology (%)	53.4	21.5	26.3

Edge count, target coverage, mean $|d|$, and inhibitory dominance are conserved within noise between annotation-selected and random features, identifying these as architectural properties of the circuit. Shared-ontology coherence attenuates from 53% to ∼24% (selection-driven) but remains 2.5–3.1× above the configuration-preserving null of 8.5%, confirming real above-chance biological coherence in random-feature circuits.

S10 Input-size normalisation detail (E5)

We define two normalisations of edge magnitude:

$$d_{\text{feature-share}} = d \cdot \frac{n_{\text{features}}}{n_{\text{ref}}}, \quad n_{\text{ref}} = 4{,}608$$

$$d_{\text{input-share}} = d \cdot \frac{n_{\text{input positions}}}{n_{\text{input ref}}}, \quad n_{\text{input ref}} = 2{,}048$$

Table S10: **Aggregate mean $|d|$ under each normalisation.**

Condition	Raw	Feature-share	Input-share
K562/K562 GF	1.050	1.050	1.050
K562/Multi GF	0.977	0.977	0.977
TS/Multi GF	0.719	0.719	0.719
scGPT TS/Multi	1.396	0.620	0.818

Paired gene-pair comparison. For 33,301 gene pairs common to both Geneformer K562/K562 and scGPT TS/Multi circuits:

- Raw scGPT / GF mean $|d|$ ratio = 1.432
- Feature-share-normalised ratio = 0.636
- Sign agreement = 0.663; Pearson $r(d_{Geneformer}, d_{scGPT}) = 0.096$

The "scGPT produces stronger effects" pattern inverts under feature-share normalisation. Per unit of SAE representational capacity, Geneformer drives larger ablation effects on shared gene pairs.

S11 Per-source-layer circuit statistics

Table S11: **Per-source-layer circuit statistics for Geneformer K562/K562.** Each source layer contributes 30 features. "Downstream layers" is the number of subsequent layers available for measurement. "Mean edges/feat." is the per-source-feature count of significant (s, t) detections summed across downstream layers (so a single long-range (s, t) pair detected at multiple downstream layers contributes multiple entries; the deduplicated circuit graph has 52,116 unique edges total, mean unique out-degree 434 per source feature).

Source layer	Downstream	Mean edges/feat. (non-dedup.)	Mean $\|d\|$	Inhibitory %
L0	17	2,459	1.17	80.5
L5	12	1,389	1.02	79.8
L11	6	1,033	0.95	80.3
L15	2	615	0.88	79.6

Attenuation. L0 features maintain more than 200 significant edges for five downstream layers before decaying. L15→L17 effects show a mild increase, consistent with late-layer consolidation of information before the output layer.

Table S12: **Per-source-layer circuit statistics for scGPT TS/Multi.**

Source layer	Downstream	Total edges	Mean $\|d\|$	Inhibitory %
L0	11	14,931	1.43	67.2
L4	7	13,067	1.46	62.8
L8	3	3,382	1.12	68.9

Note on L4 dominance. In scGPT, mid-layer L4 features carry the largest number of significant edges per feature, in contrast to Geneformer where L0 dominates. This is consistent with the attenuation profile shown in the main text and suggests that scGPT front-loads less representational work to its first layer and concentrates processing in its middle layers.
